# 4,4′,5,5′-Tetra­kis(benzyl­sulfan­yl)tetra­thia­fulvalene

**DOI:** 10.1107/S1600536811007823

**Published:** 2011-03-09

**Authors:** Cheng-Xiang Yu, Yu-Lan Zhu, Zhao-Xiang Chen, Ming-Zhu Lu, Kun Wang

**Affiliations:** a Department of Chemistry, Northeast Normal University, Changchun 130021, People’s Republic of China; b Jiangsu Key Laboratory for the Chemistry of Low-Dimensional Materials, Huaiyin Normal University, Huaian 223300, People’s Republic of China

## Abstract

The asymmetric unit of the title compound, C_34_H_28_S_8_, contains two crystallographically independent half-mol­ecules. The mol­ecules lie on centers of inversion. The four benzene rings of each mol­ecule are substantially twisted from the planes of the 1,3-dithiole rings, forming dihedral angles of 43.6 (2) and 61.4 (1)° in one mol­ecule and 54.2 (1) and 65.2 (1)° in the other.

## Related literature

For related structures, see: Abashev *et al.* (2003 ▶); Wang *et al.* (1997 ▶). For the synthesis of 4,5-bis­(3-picolyl­thio)-1,3-dithiole-2-thione, see: see: Jia *et al.* (2001 ▶). For tetra­thia­fulvalene derivatives, see: Shibaeva & Yagubskii (2004 ▶); Varma *et al.* (1987 ▶); Williams *et al.* (1984 ▶). For bond-length data, see: Allen *et al.* (1987 ▶).
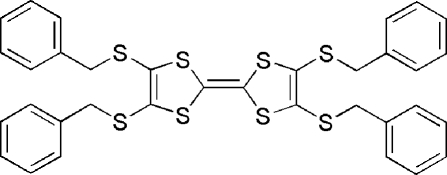


## Experimental

### 

#### Crystal data

C_34_H_28_S_8_
*M* *_r_* = 693.04
Triclinic, 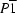

*a* = 5.7450 (7) Å
*b* = 17.052 (2) Å
*c* = 18.701 (3) Å
α = 115.199 (2)°
β = 95.238 (2)°
γ = 95.922 (2)°
*V* = 1630.1 (4) Å^3^
*Z* = 2
Mo *K*α radiation
μ = 0.57 mm^−1^
*T* = 296 K
0.3 × 0.2 × 0.1 mm

#### Data collection

Bruker SMART APEXII diffractometer
Absorption correction: multi-scan (*SADABS*; Bruker, 2000 ▶) *T*_min_ = 0.871, *T*_max_ = 0.944
11631 measured reflections
5688 independent reflections
3518 reflections with *I* > 2σ(*I*)
*R*_int_ = 0.033

#### Refinement

*R*[*F* ^2^ > 2σ(*F* ^2^)] = 0.048
*wR*(*F* ^2^) = 0.157
*S* = 1.06
5688 reflections
379 parameters
H-atom parameters constrained
Δρ_max_ = 0.32 e Å^−3^
Δρ_min_ = −0.33 e Å^−3^

### 

Data collection: *APEX2* (Bruker, 2004 ▶); cell refinement: *SAINT* (Bruker, 2004 ▶); data reduction: *SAINT*; program(s) used to solve structure: *SHELXS97* (Sheldrick, 2008 ▶); program(s) used to refine structure: *SHELXL97* (Sheldrick, 2008 ▶); molecular graphics: *ORTEP-3 for Windows* (Farrugia, 1997 ▶); software used to prepare material for publication: *SHELXL97* and *PLATON* (Spek, 2009 ▶).

## Supplementary Material

Crystal structure: contains datablocks I, New_Global_Publ_Block. DOI: 10.1107/S1600536811007823/zq2088sup1.cif

Structure factors: contains datablocks I. DOI: 10.1107/S1600536811007823/zq2088Isup2.hkl

Additional supplementary materials:  crystallographic information; 3D view; checkCIF report

## Figures and Tables

**Fig. 1.**
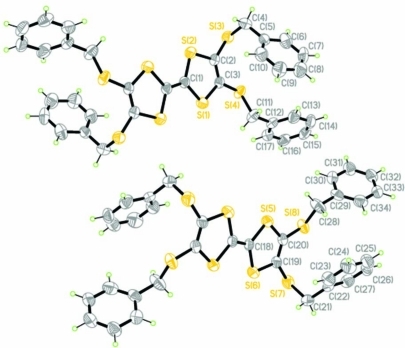
The molecular structure of the title compound with 30% probability ellipsoids.

**Fig. 2.**
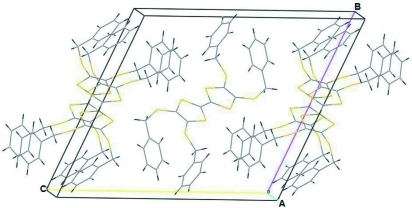
Packing diagram.

## supplementary crystallographic information

### Comment

Many researchers have focused on a particularly important class of complexes with TTF (tetrathiafulvalene) and BEDT-TTF [bis(ethylenedithio)tetrathiafulvalene] derivatives (Shibaeva & Yagubskii, 2004; Varma *et al.*, 1987). They have found related the wide range of technological applications, such as high electronic conductivity or superconductivity (Williams *et al.*, 1984). In order to obtain materials involved in nonlinear optics, opto-electronics, molecular electronics, currently, our research is focused on the synthesis and crystal structures of TTF derivatives.

The asymmetric unit of the title compound, C_34_H_28_S_8_, contains two crystallographically independent half-molecules. The molecules lie on centers of inversion. They adopt chair-like conformations and the four benzene rings of each molecule are severely twisted from the planarity of the 1,3-dithiole rings (Fig. 1). Due to the C_i_ symmetry of the molecules, the substituent groups of the TTF core are located in opposite directions, resulting in chair-like molecular conformations. The four benzene rings of each molecule are severely twisted from the planarity of the 1,3-dithiole rings. The C—S bonds in the five-membered rings fall in the range of 1.742 (4)–1.761 (4) Å and are shorter than a typical C—S single bond (1.82 Å; Allen *et al.*, 1987), revealing the high degree of conjugation into the five-membered rings of the title compound. On the other hand, the S—C(CH_2_Ph) bond distances are longer than the C-S bonds of the rings falling in the range of 1.794 (5)-1.837 (5) Å, similar to a typical C—S single bond. The mean planes of the C5–C10 and C12–C13 benzene rings [C22–C27 and C29–C34] form dihedral angles of 43.6 (2) and 61.4 (1)° [54.2 (1) and 65.2 (1)°] with the least-squares plane of the central dithiolane ring, respectively. The crystal packing diagram of the title compound is shown in Fig. 2. The shortest intermolecular S-S distances, S(1)—S(2) and S(5)—S(6) distances, are 3.793 (2) Å and 3.855 (2) Å, respectively.

### Experimental

A total of 42.15 mg (0.3 mmol) of K_2_CO_3_ was dissolved in less than 5 ml of water, and 100 mg (0.61 mmol) of 3-picolyl chloride hydrochloride was added at room temperature. After the gas evolution was stopped, a colorless dense liquid was present. Subsequently, 143.35 mg (0.15 mmol) of TBA2[Zn(DMIT)2] dissolved in 15 ml of acetonitrile was mixed with this dense liquid, and the solution was stirred at 50–60 °C for 1.5–2 h. The reaction mixture was filtered, and the solid residue was washed twice with dichloromethane (20 ml). The combined filtrate and washings were decolorized by activated charcoal. After removing the solvent, column chromatography of the crude reaction mixture on silica gel with ethyl acetate/methanol (10:1) afforded compound 1a as a yellow solid (85.5 mg, 75%). Benzyl chloride (12 ml) was added dropwise to a solution of TBA2[Zn(DMIT)2] (10 mmol) in acetone (100 ml). The mixture was refluxed under N_2_ for 24 h. Stirring was continued overnight. The resulting orange precipitate was filtered off. The product was further purified by recrystallization from methanol to give yellow needle like crystals (yield 76%). All solvent were distilled before use. 95 mg (0.25 mmol) of 4,5-bis(3-picolylthio)-1,3-dithiole-2-thione and 144 mg (0.4 mmol) of 4,5- bis(benzylthio)-1,3-dithione- 2-thione (0.3 mmol) were stirred in 30 ml of dry toluene under N_2_. Then, 2.5 ml of P(OEt)_3_ was added and the yellowish suspension was refluxed for 4 h at 120 °C. The resulting orange yellow precipitate that formed was filtered off. The red filtrate was left to stand for several days, giving pale red crystals suitable for a X-ray structure analysis.

### Refinement

All non-hydrogen atoms were located from the difference Fourier maps, and were refined anisotropically. All H atoms were positioned geometrically, and were allowed to ride on their corresponding parent atoms with *U*_iso_ = 1.2 *U*_eq_.

### Crystal data

**Table:**

C_34_H_28_S_8_	*V* = 1630.1 (4) Å^3^
*M**_r_* = 693.04	*Z* = 2
Triclinic, *P*1	*F*(000) = 720
Hall symbol: -P 1	*D*_x_ = 1.412 Mg m^−^^3^
*a* = 5.7450 (7) Å	Mo *K*α radiation, λ = 0.71073 Å
*b* = 17.052 (2) Å	θ = 1.2–25.0°
*c* = 18.701 (3) Å	µ = 0.57 mm^−^^1^
α = 115.199 (2)°	*T* = 296 K
β = 95.238 (2)°	Needle, red
γ = 95.922 (2)°	0.3 × 0.2 × 0.1 mm

### Data collection

**Table:**

Bruker SMART APEXII diffractometer	5688 independent reflections
Radiation source: fine-focus sealed tube	3518 reflections with *I* > 2σ(*I*)
graphite	*R*_int_ = 0.033
Detector resolution: 10.0 pixels mm^-1^	θ_max_ = 25.0°, θ_min_ = 1.2°
ω–scan	*h* = −6→6
Absorption correction: multi-scan (*SADABS*; Bruker, 2000)	*k* = −19→20
*T*_min_ = 0.871, *T*_max_ = 0.944	*l* = −22→19
11631 measured reflections	

### Refinement

**Table:**

Refinement on *F*^2^	Primary atom site location: structure-invariant direct methods
Least-squares matrix: full	Secondary atom site location: difference Fourier map
*R*[*F*^2^ > 2σ(*F*^2^)] = 0.048	Hydrogen site location: inferred from neighbouring sites
*wR*(*F*^2^) = 0.157	H-atom parameters constrained
*S* = 1.06	*w* = 1/[σ^2^(*F*_o_^2^) + (0.0745*P*)^2^] where *P* = (*F*_o_^2^ + 2*F*_c_^2^)/3
5688 reflections	(Δ/σ)_max_ = 0.001
379 parameters	Δρ_max_ = 0.32 e Å^−^^3^
0 restraints	Δρ_min_ = −0.33 e Å^−^^3^

### Special details

**Table:**

Geometry. All e.s.d.'s (except the e.s.d. in the dihedral angle between two l.s. planes) are estimated using the full covariance matrix. The cell e.s.d.'s are taken into account individually in the estimation of e.s.d.'s in distances, angles and torsion angles; correlations between e.s.d.'s in cell parameters are only used when they are defined by crystal symmetry. An approximate (isotropic) treatment of cell e.s.d.'s is used for estimating e.s.d.'s involving l.s. planes.
Refinement. Refinement of *F*^2^ against ALL reflections. The weighted *R*-factor *wR* and goodness of fit *S* are based on *F*^2^, conventional *R*-factors *R* are based on *F*, with *F* set to zero for negative *F*^2^. The threshold expression of *F*^2^ > σ(*F*^2^) is used only for calculating *R*-factors(gt) *etc*. and is not relevant to the choice of reflections for refinement. *R*-factors based on *F*^2^ are statistically about twice as large as those based on *F*, and *R*- factors based on ALL data will be even larger.

### Fractional atomic coordinates and isotropic or equivalent isotropic displacement parameters (Å^2^)

**Table:**

	*x*	*y*	*z*	*U*_iso_*/*U*_eq_	
S1	0.76116 (19)	0.45649 (8)	0.06508 (7)	0.0626 (3)	
S2	0.7452 (2)	0.41173 (8)	−0.10618 (7)	0.0624 (3)	
S3	0.2978 (2)	0.28449 (8)	−0.15494 (7)	0.0675 (4)	
S4	0.30556 (19)	0.33663 (8)	0.03726 (7)	0.0634 (3)	
S5	1.18791 (19)	0.39890 (7)	0.44406 (7)	0.0604 (3)	
S6	1.37020 (19)	0.51995 (7)	0.61260 (7)	0.0634 (3)	
S7	0.9859 (2)	0.44729 (8)	0.67531 (7)	0.0707 (4)	
S8	0.77397 (19)	0.31193 (7)	0.48536 (8)	0.0666 (4)	
C1	0.8989 (7)	0.4720 (3)	−0.0086 (2)	0.0521 (10)	
C2	0.5204 (7)	0.3564 (3)	−0.0793 (3)	0.0525 (10)	
C3	0.5289 (7)	0.3773 (2)	−0.0010 (3)	0.0503 (10)	
C4	0.4542 (9)	0.1914 (3)	−0.2040 (3)	0.0772 (14)	
H4A	0.3542	0.1486	−0.2525	0.093*	
H4B	0.5966	0.2120	−0.2192	0.093*	
C5	0.5204 (8)	0.1480 (3)	−0.1528 (3)	0.0588 (11)	
C6	0.3653 (9)	0.0843 (3)	−0.1473 (3)	0.0846 (16)	
H6	0.2151	0.0674	−0.1773	0.101*	
C7	0.4232 (10)	0.0452 (3)	−0.0998 (4)	0.0864 (16)	
H7	0.3148	0.0020	−0.0977	0.104*	
C8	0.6399 (11)	0.0697 (4)	−0.0556 (4)	0.0893 (16)	
H8	0.6809	0.0432	−0.0229	0.107*	
C9	0.7975 (10)	0.1321 (4)	−0.0583 (3)	0.0893 (16)	
H9	0.9465	0.1485	−0.0276	0.107*	
C10	0.7390 (8)	0.1713 (3)	−0.1062 (3)	0.0745 (14)	
H10	0.8490	0.2146	−0.1074	0.089*	
C11	0.4694 (8)	0.3179 (3)	0.1146 (3)	0.0768 (14)	
H11A	0.5860	0.2803	0.0928	0.092*	
H11B	0.5503	0.3730	0.1573	0.092*	
C12	0.2902 (7)	0.2742 (3)	0.1453 (3)	0.0576 (11)	
C13	0.2587 (10)	0.1853 (4)	0.1199 (3)	0.0823 (15)	
H13	0.3578	0.1525	0.0862	0.099*	
C14	0.0888 (10)	0.1439 (3)	0.1422 (3)	0.0807 (15)	
H14	0.0690	0.0833	0.1236	0.097*	
C15	−0.0532 (9)	0.1920 (4)	0.1922 (3)	0.0787 (15)	
H15	−0.1715	0.1637	0.2078	0.094*	
C16	−0.0264 (9)	0.2797 (4)	0.2199 (3)	0.0754 (14)	
H16	−0.1243	0.3120	0.2544	0.090*	
C17	0.1477 (9)	0.3207 (3)	0.1963 (3)	0.0734 (13)	
H17	0.1684	0.3814	0.2156	0.088*	
C18	1.4097 (7)	0.4832 (2)	0.5118 (2)	0.0515 (10)	
C19	1.1169 (7)	0.4427 (2)	0.5933 (2)	0.0524 (10)	
C20	1.0339 (7)	0.3877 (2)	0.5165 (3)	0.0519 (10)	
C21	1.1842 (9)	0.3938 (3)	0.7156 (3)	0.0731 (13)	
H21A	1.3445	0.4247	0.7265	0.088*	
H21B	1.1398	0.3982	0.7658	0.088*	
C22	1.1795 (7)	0.2990 (3)	0.6602 (2)	0.0538 (10)	
C23	1.3551 (8)	0.2731 (3)	0.6152 (3)	0.0731 (13)	
H23	1.4791	0.3147	0.6185	0.088*	
C24	1.3509 (10)	0.1866 (4)	0.5652 (3)	0.0910 (17)	
H24	1.4714	0.1696	0.5345	0.109*	
C25	1.1736 (12)	0.1262 (3)	0.5603 (3)	0.0902 (18)	
H25	1.1743	0.0673	0.5269	0.108*	
C26	0.9954 (9)	0.1488 (3)	0.6025 (3)	0.0768 (15)	
H26	0.8715	0.1065	0.5979	0.092*	
C27	0.9989 (8)	0.2348 (3)	0.6522 (3)	0.0738 (14)	
H27	0.8756	0.2508	0.6817	0.089*	
C28	0.8508 (9)	0.2203 (3)	0.4031 (3)	0.0909 (18)	
H28A	0.8838	0.2367	0.3610	0.109*	
H28B	0.9915	0.2024	0.4206	0.109*	
C29	0.6469 (8)	0.1459 (3)	0.3725 (3)	0.0634 (12)	
C30	0.4469 (9)	0.1446 (3)	0.3250 (3)	0.0723 (13)	
H30	0.4372	0.1901	0.3104	0.087*	
C31	0.2629 (8)	0.0768 (3)	0.2992 (3)	0.0754 (14)	
H31	0.1279	0.0763	0.2674	0.090*	
C32	0.2775 (9)	0.0106 (3)	0.3199 (3)	0.0768 (14)	
H32	0.1516	−0.0353	0.3024	0.092*	
C33	0.4706 (9)	0.0101 (3)	0.3652 (3)	0.0744 (14)	
H33	0.4786	−0.0358	0.3792	0.089*	
C34	0.6527 (9)	0.0760 (3)	0.3904 (3)	0.0784 (14)	
H34	0.7873	0.0743	0.4210	0.094*	

### Atomic displacement parameters (Å^2^)

**Table:**

	*U*^11^	*U*^22^	*U*^33^	*U*^12^	*U*^13^	*U*^23^
S1	0.0595 (7)	0.0670 (7)	0.0542 (7)	−0.0072 (5)	0.0140 (5)	0.0227 (6)
S2	0.0632 (7)	0.0664 (7)	0.0577 (7)	−0.0015 (6)	0.0148 (5)	0.0287 (6)
S3	0.0635 (7)	0.0725 (8)	0.0667 (8)	−0.0025 (6)	−0.0086 (6)	0.0371 (7)
S4	0.0483 (6)	0.0828 (8)	0.0765 (8)	0.0003 (6)	0.0072 (5)	0.0541 (7)
S5	0.0561 (7)	0.0603 (7)	0.0583 (7)	−0.0096 (5)	0.0001 (5)	0.0257 (6)
S6	0.0623 (7)	0.0568 (7)	0.0607 (7)	−0.0053 (5)	−0.0011 (6)	0.0214 (6)
S7	0.0860 (9)	0.0560 (7)	0.0689 (8)	0.0178 (6)	0.0295 (7)	0.0214 (6)
S8	0.0556 (7)	0.0486 (6)	0.0825 (8)	−0.0014 (5)	0.0199 (6)	0.0167 (6)
C1	0.051 (2)	0.051 (2)	0.056 (3)	0.0040 (18)	0.014 (2)	0.025 (2)
C2	0.049 (2)	0.054 (2)	0.062 (3)	0.0076 (19)	0.010 (2)	0.032 (2)
C3	0.046 (2)	0.050 (2)	0.060 (3)	0.0034 (18)	0.0080 (19)	0.029 (2)
C4	0.092 (4)	0.070 (3)	0.055 (3)	0.000 (3)	0.001 (3)	0.019 (3)
C5	0.066 (3)	0.048 (2)	0.055 (3)	0.003 (2)	0.011 (2)	0.017 (2)
C6	0.068 (3)	0.069 (3)	0.105 (4)	−0.006 (3)	0.008 (3)	0.032 (3)
C7	0.092 (4)	0.063 (3)	0.118 (5)	0.002 (3)	0.027 (4)	0.053 (3)
C8	0.097 (4)	0.082 (4)	0.103 (4)	0.027 (3)	0.025 (4)	0.050 (3)
C9	0.074 (4)	0.100 (4)	0.103 (4)	0.015 (3)	0.005 (3)	0.055 (4)
C10	0.060 (3)	0.073 (3)	0.088 (4)	−0.002 (2)	0.012 (3)	0.035 (3)
C11	0.063 (3)	0.105 (4)	0.085 (4)	0.009 (3)	0.006 (3)	0.065 (3)
C12	0.057 (3)	0.072 (3)	0.055 (3)	0.014 (2)	0.009 (2)	0.038 (2)
C13	0.104 (4)	0.085 (4)	0.081 (4)	0.036 (3)	0.042 (3)	0.047 (3)
C14	0.116 (4)	0.058 (3)	0.083 (4)	0.019 (3)	0.038 (3)	0.040 (3)
C15	0.085 (4)	0.096 (4)	0.079 (4)	0.006 (3)	0.020 (3)	0.060 (3)
C16	0.086 (4)	0.094 (4)	0.063 (3)	0.032 (3)	0.032 (3)	0.043 (3)
C17	0.091 (4)	0.065 (3)	0.063 (3)	0.012 (3)	0.007 (3)	0.028 (3)
C18	0.054 (2)	0.044 (2)	0.060 (3)	0.0030 (18)	0.001 (2)	0.028 (2)
C19	0.057 (2)	0.043 (2)	0.057 (3)	0.0114 (19)	0.011 (2)	0.021 (2)
C20	0.052 (2)	0.043 (2)	0.061 (3)	0.0092 (18)	0.011 (2)	0.023 (2)
C21	0.101 (4)	0.065 (3)	0.049 (3)	0.011 (3)	0.011 (2)	0.021 (2)
C22	0.064 (3)	0.060 (3)	0.041 (2)	0.008 (2)	0.005 (2)	0.027 (2)
C23	0.066 (3)	0.073 (3)	0.076 (3)	0.003 (3)	0.017 (3)	0.030 (3)
C24	0.092 (4)	0.086 (4)	0.089 (4)	0.035 (3)	0.029 (3)	0.026 (3)
C25	0.104 (5)	0.059 (3)	0.092 (4)	0.009 (3)	−0.021 (4)	0.026 (3)
C26	0.067 (3)	0.064 (3)	0.096 (4)	−0.017 (3)	−0.010 (3)	0.043 (3)
C27	0.062 (3)	0.093 (4)	0.076 (3)	0.003 (3)	0.011 (2)	0.048 (3)
C28	0.074 (3)	0.074 (3)	0.082 (4)	−0.020 (3)	0.026 (3)	−0.001 (3)
C29	0.060 (3)	0.060 (3)	0.049 (3)	−0.004 (2)	0.015 (2)	0.006 (2)
C30	0.084 (3)	0.060 (3)	0.077 (3)	0.009 (3)	0.009 (3)	0.035 (3)
C31	0.061 (3)	0.066 (3)	0.088 (4)	0.003 (2)	−0.008 (3)	0.028 (3)
C32	0.075 (3)	0.059 (3)	0.080 (4)	−0.006 (2)	0.001 (3)	0.021 (3)
C33	0.089 (4)	0.053 (3)	0.074 (3)	0.001 (3)	−0.005 (3)	0.027 (3)
C34	0.074 (3)	0.079 (4)	0.069 (3)	0.012 (3)	−0.004 (3)	0.022 (3)

### Geometric parameters (Å, °)

**Table:**

S1—C3	1.742 (4)	C13—H13	0.9300
S1—C1	1.750 (4)	C14—C15	1.356 (7)
S2—C2	1.756 (4)	C14—H14	0.9300
S2—C1	1.757 (4)	C15—C16	1.344 (7)
S3—C2	1.735 (4)	C15—H15	0.9300
S3—C4	1.837 (5)	C16—C17	1.372 (6)
S4—C3	1.745 (4)	C16—H16	0.9300
S4—C11	1.813 (4)	C17—H17	0.9300
S5—C20	1.749 (4)	C18—C18^ii^	1.333 (7)
S5—C18	1.752 (4)	C19—C20	1.344 (6)
S6—C19	1.757 (4)	C21—C22	1.499 (6)
S6—C18	1.761 (4)	C21—H21A	0.9700
S7—C19	1.745 (4)	C21—H21B	0.9700
S7—C21	1.827 (5)	C22—C23	1.360 (6)
S8—C20	1.744 (4)	C22—C27	1.376 (6)
S8—C28	1.794 (5)	C23—C24	1.366 (7)
C1—C1^i^	1.343 (7)	C23—H23	0.9300
C2—C3	1.347 (6)	C24—C25	1.339 (7)
C4—C5	1.486 (6)	C24—H24	0.9300
C4—H4A	0.9700	C25—C26	1.337 (7)
C4—H4B	0.9700	C25—H25	0.9300
C5—C6	1.377 (6)	C26—C27	1.358 (7)
C5—C10	1.378 (6)	C26—H26	0.9300
C6—C7	1.356 (7)	C27—H27	0.9300
C6—H6	0.9300	C28—C29	1.505 (6)
C7—C8	1.349 (7)	C28—H28A	0.9700
C7—H7	0.9300	C28—H28B	0.9700
C8—C9	1.345 (7)	C29—C34	1.371 (7)
C8—H8	0.9300	C29—C30	1.379 (6)
C9—C10	1.365 (7)	C30—C31	1.366 (6)
C9—H9	0.9300	C30—H30	0.9300
C10—H10	0.9300	C31—C32	1.348 (6)
C11—C12	1.503 (6)	C31—H31	0.9300
C11—H11A	0.9700	C32—C33	1.336 (6)
C11—H11B	0.9700	C32—H32	0.9300
C12—C17	1.357 (6)	C33—C34	1.340 (6)
C12—C13	1.368 (6)	C33—H33	0.9300
C13—C14	1.345 (6)	C34—H34	0.9300
			
C3—S1—C1	95.58 (19)	C17—C16—H16	120.5
C2—S2—C1	95.26 (19)	C12—C17—C16	121.1 (5)
C2—S3—C4	100.3 (2)	C12—C17—H17	119.4
C3—S4—C11	102.9 (2)	C16—C17—H17	119.4
C20—S5—C18	95.82 (19)	C18^ii^—C18—S5	122.4 (4)
C19—S6—C18	95.26 (18)	C18^ii^—C18—S6	123.4 (4)
C19—S7—C21	101.0 (2)	S5—C18—S6	114.2 (2)
C20—S8—C28	102.0 (2)	C20—C19—S7	125.4 (3)
C1^i^—C1—S1	122.5 (4)	C20—C19—S6	117.4 (3)
C1^i^—C1—S2	122.9 (4)	S7—C19—S6	117.1 (2)
S1—C1—S2	114.5 (2)	C19—C20—S8	123.9 (3)
C3—C2—S3	125.7 (3)	C19—C20—S5	117.2 (3)
C3—C2—S2	116.9 (3)	S8—C20—S5	118.8 (2)
S3—C2—S2	117.3 (2)	C22—C21—S7	113.2 (3)
C2—C3—S1	117.6 (3)	C22—C21—H21A	108.9
C2—C3—S4	123.0 (3)	S7—C21—H21A	108.9
S1—C3—S4	119.1 (2)	C22—C21—H21B	108.9
C5—C4—S3	113.3 (3)	S7—C21—H21B	108.9
C5—C4—H4A	108.9	H21A—C21—H21B	107.8
S3—C4—H4A	108.9	C23—C22—C27	117.2 (4)
C5—C4—H4B	108.9	C23—C22—C21	120.8 (4)
S3—C4—H4B	108.9	C27—C22—C21	122.0 (4)
H4A—C4—H4B	107.7	C22—C23—C24	120.6 (5)
C6—C5—C10	116.2 (5)	C22—C23—H23	119.7
C6—C5—C4	122.2 (4)	C24—C23—H23	119.7
C10—C5—C4	121.5 (4)	C25—C24—C23	120.1 (5)
C7—C6—C5	122.6 (5)	C25—C24—H24	120.0
C7—C6—H6	118.7	C23—C24—H24	120.0
C5—C6—H6	118.7	C26—C25—C24	121.3 (5)
C8—C7—C6	119.2 (5)	C26—C25—H25	119.3
C8—C7—H7	120.4	C24—C25—H25	119.3
C6—C7—H7	120.4	C25—C26—C27	118.7 (5)
C9—C8—C7	120.7 (6)	C25—C26—H26	120.7
C9—C8—H8	119.7	C27—C26—H26	120.7
C7—C8—H8	119.7	C26—C27—C22	122.0 (5)
C8—C9—C10	120.1 (5)	C26—C27—H27	119.0
C8—C9—H9	120.0	C22—C27—H27	119.0
C10—C9—H9	120.0	C29—C28—S8	108.6 (3)
C9—C10—C5	121.3 (5)	C29—C28—H28A	110.0
C9—C10—H10	119.3	S8—C28—H28A	110.0
C5—C10—H10	119.3	C29—C28—H28B	110.0
C12—C11—S4	106.2 (3)	S8—C28—H28B	110.0
C12—C11—H11A	110.5	H28A—C28—H28B	108.3
S4—C11—H11A	110.5	C34—C29—C30	117.1 (4)
C12—C11—H11B	110.5	C34—C29—C28	121.2 (5)
S4—C11—H11B	110.5	C30—C29—C28	121.7 (5)
H11A—C11—H11B	108.7	C31—C30—C29	120.3 (5)
C17—C12—C13	117.8 (4)	C31—C30—H30	119.9
C17—C12—C11	121.4 (4)	C29—C30—H30	119.9
C13—C12—C11	120.7 (4)	C32—C31—C30	119.9 (5)
C14—C13—C12	121.9 (5)	C32—C31—H31	120.1
C14—C13—H13	119.0	C30—C31—H31	120.1
C12—C13—H13	119.0	C33—C32—C31	120.8 (5)
C13—C14—C15	118.9 (5)	C33—C32—H32	119.6
C13—C14—H14	120.5	C31—C32—H32	119.6
C15—C14—H14	120.5	C32—C33—C34	119.9 (5)
C16—C15—C14	121.3 (5)	C32—C33—H33	120.1
C16—C15—H15	119.4	C34—C33—H33	120.1
C14—C15—H15	119.4	C33—C34—C29	122.1 (5)
C15—C16—C17	118.9 (5)	C33—C34—H34	119.0
C15—C16—H16	120.5	C29—C34—H34	119.0
			
C3—S1—C1—C1^i^	−178.8 (5)	C20—S5—C18—C18^ii^	177.2 (5)
C3—S1—C1—S2	3.6 (3)	C20—S5—C18—S6	−3.6 (3)
C2—S2—C1—C1^i^	178.7 (5)	C19—S6—C18—C18^ii^	−177.2 (5)
C2—S2—C1—S1	−3.7 (3)	C19—S6—C18—S5	3.6 (3)
C4—S3—C2—C3	−108.3 (4)	C21—S7—C19—C20	104.9 (4)
C4—S3—C2—S2	75.8 (3)	C21—S7—C19—S6	−78.2 (3)
C1—S2—C2—C3	2.3 (4)	C18—S6—C19—C20	−2.2 (4)
C1—S2—C2—S3	178.6 (2)	C18—S6—C19—S7	−179.4 (2)
S3—C2—C3—S1	−176.1 (2)	S7—C19—C20—S8	1.4 (6)
S2—C2—C3—S1	−0.2 (5)	S6—C19—C20—S8	−175.4 (2)
S3—C2—C3—S4	−2.2 (6)	S7—C19—C20—S5	176.9 (2)
S2—C2—C3—S4	173.7 (2)	S6—C19—C20—S5	0.1 (5)
C1—S1—C3—C2	−2.1 (4)	C28—S8—C20—C19	−143.4 (4)
C1—S1—C3—S4	−176.2 (2)	C28—S8—C20—S5	41.1 (3)
C11—S4—C3—C2	141.7 (4)	C18—S5—C20—C19	2.1 (4)
C11—S4—C3—S1	−44.4 (3)	C18—S5—C20—S8	177.9 (2)
C2—S3—C4—C5	65.9 (4)	C19—S7—C21—C22	−65.1 (4)
S3—C4—C5—C6	84.5 (5)	S7—C21—C22—C23	101.7 (5)
S3—C4—C5—C10	−93.5 (5)	S7—C21—C22—C27	−78.0 (5)
C10—C5—C6—C7	−0.9 (8)	C27—C22—C23—C24	−0.7 (7)
C4—C5—C6—C7	−179.0 (5)	C21—C22—C23—C24	179.6 (4)
C5—C6—C7—C8	0.5 (9)	C22—C23—C24—C25	−0.3 (8)
C6—C7—C8—C9	0.0 (9)	C23—C24—C25—C26	1.3 (9)
C7—C8—C9—C10	0.0 (9)	C24—C25—C26—C27	−1.2 (8)
C8—C9—C10—C5	−0.4 (8)	C25—C26—C27—C22	0.2 (7)
C6—C5—C10—C9	0.9 (7)	C23—C22—C27—C26	0.8 (7)
C4—C5—C10—C9	179.0 (5)	C21—C22—C27—C26	−179.5 (4)
C3—S4—C11—C12	−174.6 (3)	C20—S8—C28—C29	176.3 (4)
S4—C11—C12—C17	−77.4 (5)	S8—C28—C29—C34	−103.0 (5)
S4—C11—C12—C13	99.8 (5)	S8—C28—C29—C30	77.3 (5)
C17—C12—C13—C14	1.9 (7)	C34—C29—C30—C31	1.4 (7)
C11—C12—C13—C14	−175.4 (5)	C28—C29—C30—C31	−178.9 (4)
C12—C13—C14—C15	−0.9 (8)	C29—C30—C31—C32	−0.4 (8)
C13—C14—C15—C16	−0.2 (8)	C30—C31—C32—C33	−0.3 (8)
C14—C15—C16—C17	0.3 (8)	C31—C32—C33—C34	−0.1 (8)
C13—C12—C17—C16	−1.7 (7)	C32—C33—C34—C29	1.3 (8)
C11—C12—C17—C16	175.5 (4)	C30—C29—C34—C33	−1.9 (7)
C15—C16—C17—C12	0.7 (7)	C28—C29—C34—C33	178.4 (4)

Symmetry codes: (i) −*x*+2, −*y*+1, −*z*; (ii) −*x*+3, −*y*+1, −*z*+1.
